# Method for CaOx crystals isolation from plant leaves

**DOI:** 10.1016/j.mex.2022.101798

**Published:** 2022-07-28

**Authors:** Ivan T. Cerritos-Castro, Araceli Patrón-Soberano, Ana P. Barba de la Rosa

**Affiliations:** IPICYT, Instituto Potosino de Investigación Científica y Tecnológica A.C., San Luis Potosí, San Luis Potosí, 78216 México

**Keywords:** Amaranth, Biomineralization, Calcium oxalate crystals, Heavy liquids, Microscopy

## Abstract

Although calcium oxalate (CaOx) crystals are present in many plants they are poorly studied. A possible limitation is the lack of methods for CaOx crystals isolation at high concentration and high purity, which is required for the analysis of their associated biomolecules such as proteins. To our knowledge, there are only four works that have isolated proteins from CaOx crystals. Those methods basically consist of grinding the plant material, filtration steps, enzymatic digestions, and density-based separation. However, they lack of steps to evaluate the quality and purity of the isolated crystals. Likewise, those works do not evaluate whether the crystals obtained carry contaminating proteins.

In the present work a detailed method for CaOx crystals isolation from amaranth leaves is described, which can be used to isolate crystals from other plant leaves. The present method is based on previous works with the addition of cleaning steps to removal contaminating protein, separation of crystals by size, and microscopic monitoring to validate the purification efficiency.

Main steps for CaOx crystals isolation:

• Plant leaves are ground and several washing steps, including enzymatic digestions and centrifugation, are carried out to remove cellular debris and contaminating proteins.
• CaOx crystals are enriched by centrifugation in sodium polytungstate.
• The different forms of crystals are separated by filtration.

## Graphical Abstract

**Figure:**
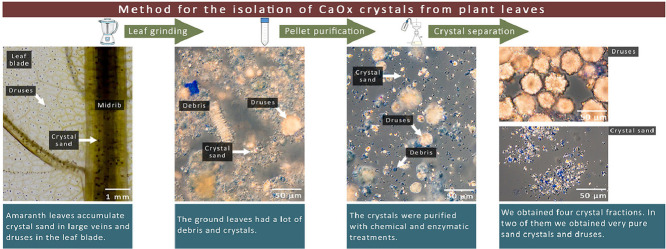

Specifications table

**Table:** 

Subject Area;	Biochemistry, Genetics, and Molecular Biology
More specific subject area;	Plant histology, microscopy
Method name;	Method for CaOx crystals isolation from plant leaves
Name and reference of original method;	X. Li, D. Zhang, V.J. Lynch-Holm, T.W. Okita, V.R. Franceschi, Isolation of a crystal matrix protein associated with calcium oxalate precipitation in vacuoles of specialized cells. Plant Physiol. 133 (2003) 549–59. https://doi.org/10.1104/pp.103.023556. D. Jáuregui-Zúñiga, J.P. Reyes-Grajeda, A. Moreno, Modifications on the morphology of synthetically-grown calcium oxalate crystals by crystal-associated proteins isolated from bean seed coats (*Phaseolus vulgaris*). Plant Sci. 168 (2005) 1163–1169. https://doi.org/10.1016/j.plantsci.2004.12.013
Resource availability;	Does not apply

## Reagents and Equipment

Macerozyme Cat. No. M8002 (Goldbio, St. Louis, Mo, USA)

Driselase Cat. No. D8037 (Sigma-Aldrich, St. Louis, Mo, USA)

Pancreatin Cat. No. P3292 (Sigma-Aldrich, St. Louis, Mo, USA)

Sodium polytungstate (3Na_2_WO_4_•9WO_3_•H_2_O), (Fluka, Seelze, BS, Germany)

Blender Oster BLSTFG-C00-013 (Oster-sunbeam, Boca Raton, FL, USA),

Centrifuge Avanti J-26S XPI (Beckman Coulter, Brea, USA)

Incubation chamber LM-5002 (MRC Laboratory Equipment)

Thermomixer compact 5350 (Eppendorf. Hamburg, Germany)

Vacuum filtration system Sterifil (Millipore, Burlington, USA)

Vacuum pump Xx5500000 (Millipore, Burlington, USA)

Ultrasonic cleaner 2510R-MTH (Bransonic, Danbury, USA)

Zeiss Axio imager M2 microscope (Carl-Zeiss, Oberkochen, Germany)

## Method details

**Fig. 1:**
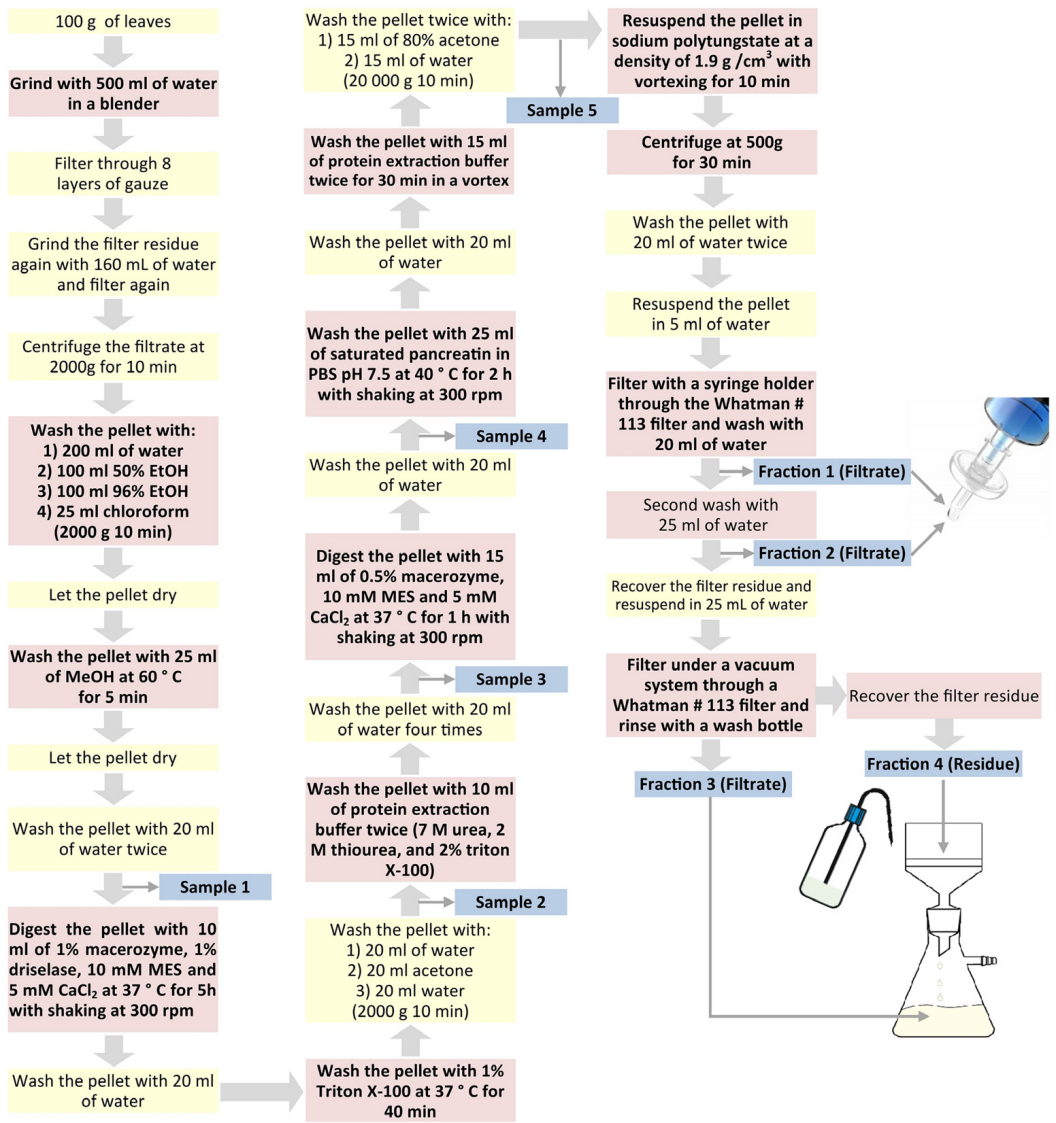
Schematic representation of the CaOx crystal isolation.

Method workflow is described in Fig. 1 for easy reference

### Crystal isolation

1. Fresh plant leaves (100 g) are washed with tap water and ground with 500 mL of deionized water (DW) in a blender at maximum speed for 2 to 3 min until no large leaf fragments are observed.
2. Filter through 8 layers of gauze and grind the residues with 160 mL of DW for 2 min and filter again in new 8 layers of gauze. The filtrate is centrifuge at 2000 g for 10 min at room temperature. Supernatants are discarded and the pellet is washed with 200 mL of DW.
3. For lipids removal, wash the pellet with 100 mL of 50% EtOH, 100 mL of 96% EtOH, 25 mL of chloroform, centrifuge and allow to dry at room temperature.
4. For chlorophyll removal, wash the pellet with 25 mL of MeOH at 60 °C for 5 min, centrifuge and allow to dry. Pellet is washed twice with 20 mL of DW and an aliquot (10 µL) is taken and named as **Sample 1**.
5. To remove cell wall debris, pellet is digested with 10 mL of cell wall digestion solution (macerozyme 1%, driselase, 10 mM MES, and 5 mM CaCl_2_) at 37 °C for 5 h with constant shaking at 300 r.p.m.
6. To remove contaminating proteins (from the leaves themselves and the cell wall digestion solution), wash the pellet with DW followed by a solution containing 1% Triton X-100 for 40 min at 37 °C. To remove Triton X-100, wash the pellet with 20 mL of DW and acetone. At this point, a second aqueous aliquot (10 µL) is taken (**Sample 2)**.
7. To remove strongly adhered proteins, wash the pellet with 10 mL of protein extraction buffer (7 M urea, 2 M thiourea, and 2% Triton X-100), mix with vortex for 1 min, centrifuge and wash the pellet four times with 20 mL of DW. At this point the third aliquot (10 µL) is taken (**Sample 3)**.
8. If cellular debris are still present, digest the pellet with 25 mL of solution containing 0.5% macerozyme in 10 mM MES pH 5, 5 mM CaCl_2_ at 37 °C for 1 h with constant shaking at 300 r.p.m., Centrifuge, and wash the pellet with 20 mL of DW. The fourth aliquot (10 µL) is taken (**Sample 4)**.
9. To hydrolyze persistent proteins, pellet is digested with 25 mL of saturated pancreatin in PBS 140 mM pH 7.5 at 40 °C for 2 h with constant shaking at 300 r.p.m. Centrifuge and wash the pellet with DW, followed by 15 mL of protein extraction buffer for 30 min mixing with vortex. Centrifuge again and wash the pellet with 15 mL of 80% acetone and 15 mL of DW twice. The fifth aqueous aliquot (10 µL) is taken (**Sample 5)**.
10. Resuspend the pellet in 10 mL of sodium polytungstate at 1.9 g/cm^3^ density and mix with vortex for 10 min and centrifuge at 500 g for 30 min at room temperature. Carefully remove the upper organic phase with a micropipette and then decant the rest avoiding loss of the pellet.
11. To eliminate sodium polytungstate, wash the pellet with 20 mL of DW twice and resuspende in 5 mL of fresh DW. Suspension is filtered through a Whatman #113 filter paper (pore size of 30 µm) using a syringe filter holder. This filtrate is identified as **Fraction 1**.
12. Wash the sediment from the filter holder by passing 25 mL of DW with shaking. This filtrate is identified as **Fraction 2**.
13. Open the filter holder and recover the sediment by washing with 25 mL of DW and vacuum filter through a Whatman #113 filter. Wash the sediment with gentle stream of DW (around 25 mL). The filtrate is identified as **Fraction 3** and the residue on the filter paper as **Fraction 4**
14. Centrifuge all fractions, discard supernatants, and allow crystals to dry and save for future analyses.

### Method monitoring

Samples and Fractions collected were analyzed to evaluate the effectiveness of the method in removing debris and contaminant proteins. They were centrifuged, resuspended in 20 µL of DW, and stained with 2 µL of Coomassie solution (0.05% Coomassie brilliant blue R-250, 40% MeOH, and 10% acetic acid) for 3 min. Samples were centrifuged, supernatants were discarded, and pellets were washed with 100 µL of distaining solution (20% MeOH, 5% acetic acid) for 25 min, followed by a wash with DW. Pellets were resuspended in 40% glycerol, and a drop of each sample was mounted on glass slides and observed under Differential Interference Contrast (DIC) microscopy.

*Amaranthus cruentus* accumulates two kinds of CaOx crystals shapes: crystal sand and druses. Crystal sand consist of individual crystals with semi-pyramid shape with sizes around 3 µm. On the other hand, druses consist of semi-spherical conglomerates of multiple crystals with sizes around 50 µm. Both types of crystals shine under polarized light, making them easy to distinguish from cellular debris when observed under DIC microscopy. Besides, Coomassie stain proteins allowing contrasting cellular debris.

**Fig. 2:**
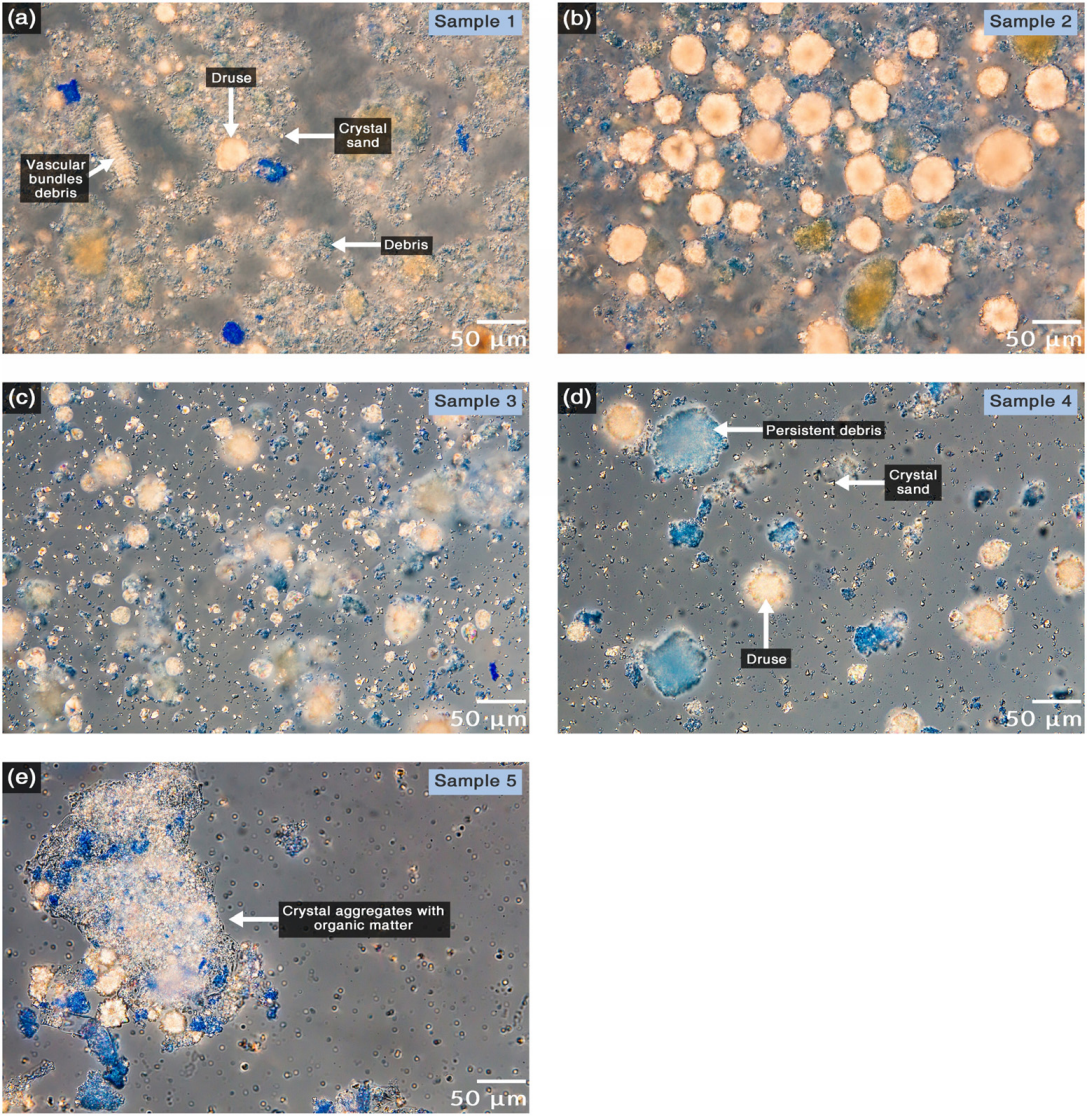
Microscopy monitoring of the CaOx crystal isolation. DIC microscopy images of samples took along the isolation process stained with Coomassie brilliant blue. (a) Ground leaves after filtration in gauze and organic solvent treatment. Chlorophyll was eliminated, and large debris remained like xylem vessels (vascular bundles debris). (b) Pellet after first digestion with cell wall digestion enzymes and first protein extraction with triton x-100. Large debris were eliminated; however, there are still much small debris with some protein content (stains blue). (c) Pellet after the first treatment with protein extraction buffer. We did not observe a significant change in debris staining. (d) Pellet after second digestion with cell wall digestion enzyme. We observed a slight reduction in the debris amount at the time that this commenced to aggregate. (e) Pellet after pancreatin digestion and a second protein extraction treatment. We observed enrichment in the concentration of crystals and that these were added together with the debris.

The chlorophyll removal treatment was effective since from Sample 1 all the green color was lost. However, this sample still contained cell debris with proteins and remains of vascular bundles (Fig. 2a). After the first treatment with macerozyme, a decrease of cellular debris and no more vascular bundles were observed (Fig. 2b), but even after the first protein extraction treatment, contaminating proteins were still observed (Figure 2c). After the second treatment with macerozyme it was observed that debris started to form aggregates (Fig. 2d), then a digestion treatment with pancreatin (a broad-spectrum protease, lipase, and amylase) was carried out, followed by a strong protein extraction step using chaotropic agents. Although a decrease in debris content was observed, there were still some that persisted as observed by blue staining and they tended to aggregates with crystal sand (Fig. 2e). This aggregates formation could be due to the fact that the washing processes exposed the crystal charges promoting them to stick together and with the remaining debris [1].

**Fig. 3:**
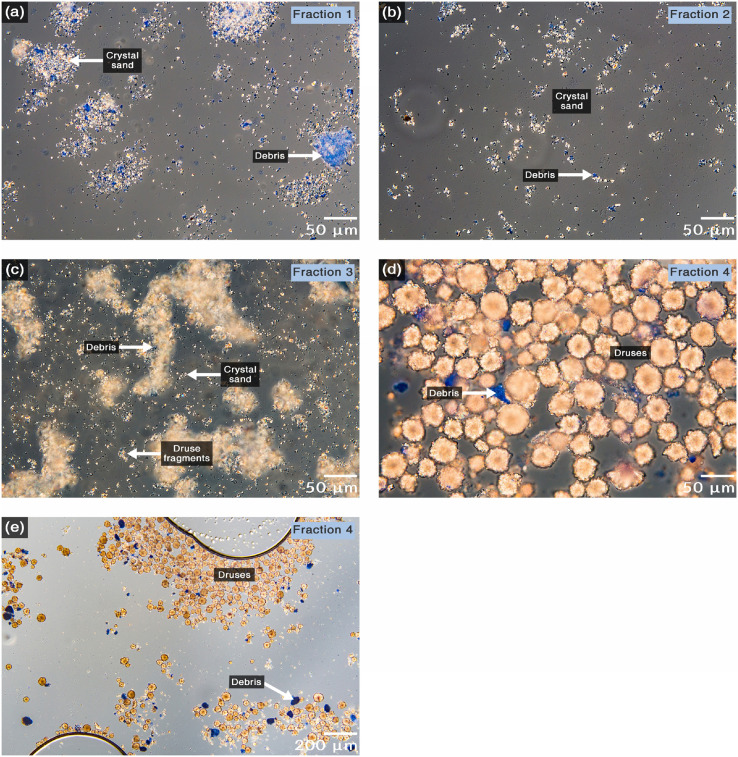
Microscopy monitoring of the CaOx crystal purification. DIC microscopy images of samples took along the purification process and stained with Coomassie brilliant blue. (a) Fraction obtained after the first filtration in the filter holder. This fraction consisted of almost only crystal sand and some debris. (b) Fraction obtained after the second wash in the filter holder. This fraction was similar to the previous but less concentrated. (c) Fraction obtained after filtration in the vacuum filtration system. This fraction was the most concentrated in crystals and with little debris content. It had a lot of crystal sand but also druse fragments which were broken during the isolation process. (d and e) Fraction remained in the residue of the vacuum filter with high and low magnification, respectively. This fraction consisted of almost only druses and some debris that were retained by the filter. Almost all crystal sand and druse fragments were effectively washed out of the pellet with a pore size of 30 µm.

Centrifugation with sodium polytungstate is an essential step in which crystals are separated from debris by density differences. CaOx crystals have a density around 1.84 to 2.04 g/cm^3^, depending on their protein content [2]. On the other hand, subcellular structures range from approximately 1.19 g/cm^3^ for the endoplasmic reticulum until 1.75 g/cm^3^ for nuclei acids and ribosomes [3]. Hence, sodium polytungstate at 1.9 g/cm^3^ should separate the high-density crystals from cellular debris. This process effectively removes most cellular debris, the few that remain were eliminated in the filtration steps. The Whatman filter #113 has a pore size of 30 µm, crystal sand has a size around 3 µm, druses around 50 µm, and most debris reached sizes larger than 30 µm after their aggregation (Fig. 2d). Thus, the first syringe holder filtrate (Fraction 1) consisted almost only of crystal sand and some debris (Fig 3a). The second wash of the residue on the syringe holder (Fraction 2) was very similar to Fraction 1 but less concentrated (Fig. 3b). When the residue of the syringe was filtered through the same filter number, but on a vacuum filtration system, the filtrate (Fraction 3) consisted of a very concentrated sample with crystal sand, a lot of druses fragments, and some debris (Fig 3c). Finally, the residue (Fraction 4) consisted mostly of druses and some debris (Fig. 3d,e).

At the end of the process, three Fractions enriched in CaOx crystals with different amounts of impurities were obtained. Debris may be composed of recalcitrant compounds as they endured three digestion steps. A major concern about CaOx crystals isolation and their associated proteins is the cross-contamination. Persistent debris appeared to have proteins (stained blue) that could cross-contaminate the samples. However, at the end of purification, debris were few compared to crystal concentration, the vast majority of them were retained in filter in Fraction 4 (Fig. 3e). In particular, Fraction 3 (Fig. 3c) could be useful for future studies directed to protein identification.

The present method provides outstanding results to isolate CaOx crystals and is a good start point for the isolation of their associated proteins, but it has three issues that could be improve. The first is that the most enriched sample in crystals, Fraction 3, consisted of a mixture of crystal sand and druses fragments. The second point is take attention in the filter pore and identifies the optimum for each specific crystal shape. Finally, although the remaining debris did not affect the purposes of crystal purification, perhaps other studies (like structural studies) require a sample with higher purity, and then it is worth to try digestions with other stronger enzymes able to degrade recalcitrant matter.

## Additional information

### Background

Calcium oxalate (CaOx) is present in most photosynthetic plants and represents up to 80% of dry biomass [4]. Unlike amorphous chemically obtained CaOx crystals, plant crystals have particular shapes [5]. Crystal morphologies include prismatic crystals, styloids, raphides, crystal sand, and druses, and these types accumulate in a species- and tissue-specific manner [4]. It is hypothesized that specific proteins are responsible for their peculiar shapes [5]. In fact, in previous works some proteins have been isolated from CaOx crystals and their nucleation capacity has been tested; however, those proteins have not been identified [6], [7], [8], [9].

Several hypotheses have been proposed about the function of CaOx crystals with calcium homeostasis being the most widely accepted. However, previous work has shown that eliminating the ability to form CaOx crystals in *Medicago truncatula* (a species that accumulates CaOx crystals) the plant growth was not affected [10].

Thus, despite its wide presence and potential importance in plant physiology, CaOx crystals are poorly understood. Isolating and identifying the proteins associated with CaOx crystal could be valuable to understand their functions and the mechanisms responsible for their particular shapes. However, there is few work on this topic. One of the possible reasons is the lack of methods and strategies for plant CaOx crystals isolation and identification of their associated proteins. To our knowledge, only four works have isolated proteins bound to CaOx crystals. However, they do not evaluate the quality and purity of the isolated crystals and whether the crystals carry contaminating proteins [6], [7], [8], [9].

Here, a new method for CaOx crystals isolation in which the efficiency to remove contaminating proteins and cellular debris is evaluated. This method is based on previous works [6], [7], [8], [9], but several steps and process monitoring were added. It is worth emphasizing that obtaining CaOx crystals at high purity is very difficult as persistent debris remains along the process. However, after multiple purification steps, it was possible to obtain a sample enriched in crystals with a low concentration of debris and contaminating proteins.

## Declaration of interests

The authors declare that they have no known competing financial interests or personal relationships that could have appeared to influence the work reported in this paper.

## Data Availability

Data will be made available on request.
